# A hot white dwarf merger remnant revealed by an ultraviolet detection of carbon

**DOI:** 10.1038/s41550-025-02590-y

**Published:** 2025-08-06

**Authors:** Snehalata Sahu, Antoine Bédard, Boris T. Gänsicke, Pier-Emmanuel Tremblay, Detlev Koester, Jay Farihi, J. J. Hermes, Mark A. Hollands, Tim Cunningham, Seth Redfield

**Affiliations:** 1 Department of Physics, University of Warwick, Coventry, UK; 2 Institut für Theoretische Physik und Astrophysik, University of Kiel, Kiel, Germany; 3 Department of Physics & Astronomy, University College London, London, UK; 4 Department of Astronomy & Institute for Astrophysical Research, Boston University, Boston, MA USA; 5 Center for Astrophysics, Harvard and Smithsonian, Cambridge, MA USA; 6 Astronomy Department and Van Vleck Observatory, Wesleyan University, Middletown, CT USA

**Keywords:** Stars, Astronomy and astrophysics

## Abstract

Atmospheric carbon has been detected in the optical spectra of six hydrogen-rich ultra-massive white dwarfs, revealing large carbon abundances (log(C/H) > −0.5) attributable to the convective dredge-up of internal carbon into thin hydrogen surface layers. These rare white dwarfs likely originate from stellar mergers, making them ‘smoking guns’ for one of the binary evolution channels leading to thermonuclear supernovae. However, optical spectroscopy can uncover only the most carbon-enriched objects, suggesting that many more merger remnants may masquerade as normal pure-hydrogen-atmosphere white dwarfs. Here we report the discovery of atmospheric carbon in a Hubble Space Telescope far-ultraviolet spectrum of WD 0525+526, a long-known hydrogen-rich ultra-massive white dwarf. The carbon abundance (log(C/H) = −4.62) is 4–5 dex lower than in the six counterparts and thus detectable only at ultraviolet wavelengths. We find that the total masses of hydrogen and helium in the envelope (10^−13.8^ and 10^−12.6^ of the total white dwarf mass, respectively) are substantially lower than those expected from single-star evolution, implying that WD 0525+526 is a merger remnant. Our modelling indicates that the low surface carbon abundance arises from an envelope structure in which a thin hydrogen-rich layer floats atop a semi-convection zone—a process that has been largely overlooked in white dwarfs. Our study highlights the importance of ultraviolet spectroscopy in identifying and characterizing merger remnants.

## Abstract (web summary)

Ultraviolet observations of an ultra-massive white dwarf with the Hubble Space Telescope reveal small amounts of carbon on the surface, identifying it as a merger remnant.

## Main

**Fig. 1:**
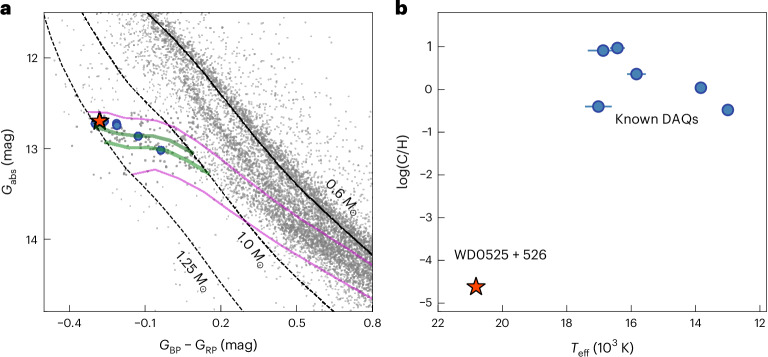
DAQ white dwarfs in the Gaia Hertzsprung–Russell diagram and the distribution of their carbon abundances. WD 0525+526 is indicated by a red star, while the six published white dwarfs with spectral type DAQ^8–10^ are shown as blue circles. **a**, Gaia Hertzsprung–Russell diagram showing the DAQ stars and white dwarfs lying within 100 pc (ref. ^17^; grey dots) where *G*_abs_ (absolute), *G*_BP_ and *G*_RP_ are the magnitudes in Gaia filters. The cooling track of a standard 0.6 *M*_⊙_ hydrogen-atmosphere white dwarf^38^ is overlaid as a solid black line, and similar cooling tracks for 1.0 and 1.25 *M*_⊙_ white dwarfs with thinner hydrogen and helium envelope layers^5^ are displayed as dashed black lines. The solid magenta curves indicate where standard white dwarf models (without distillation) have crystallized mass fractions of 20% (top curve) and 80% (bottom curve)^38^. The solid green curves show the beginning (top curve) and end (bottom curve) of the distillation process triggered by crystallization in ultra-massive white dwarf models^5^. We note that five DAQ stars are slightly brighter than the predicted onset of distillation; this is because the models displayed here assume a pure-hydrogen atmosphere and fixed envelope layer masses, while the DAQ white dwarfs contain atmospheric carbon and have different envelope structures. In particular, the redistribution of flux from ultraviolet to optical wavelengths by carbon lines makes white dwarfs brighter in Gaia *G*_abs_. **b**, Photospheric carbon abundances of the DAQ stars as a function of effective temperature (*T*_eff_) with 1*σ* error bars for the measurements. The carbon abundance of WD 0525+526 is several orders of magnitude lower than those of the previously known cooler DAQ white dwarfs and could only be detected in the HST far-UV spectrum.

The spacecraft Gaia has revealed the detailed structure of white dwarf cooling tracks in the Hertzsprung–Russell diagram^1^, uncovering a feature known as the Q-branch, an overdensity associated with white dwarfs of all masses that experience a cooling delay of about 1 Gyr owing to core crystallization and related physical processes^2^ (Fig. 1). The ultra-massive (≥1.1 solar masses (*M*_⊙_)) members of the Q-branch were found to have large space velocity dispersions indicative of abnormally old ages, implying that 5–9% of all ultra-massive white dwarfs experience an additional cooling delay of at least 8 Gyr (ref. ^3^). The leading explanation for this extra delay is the release of gravitational energy through the solid–liquid distillation of ^22^Ne triggered by crystallization in white dwarfs with carbon–oxygen cores^4,5^. This subpopulation of delayed white dwarfs is interpreted as being the descendants of certain types of stellar mergers, such as the merger of a white dwarf with a subgiant star^6^. However, it is challenging to unambiguously attribute a merger history to individual ultra-massive white dwarfs, as their core compositions (carbon–oxygen or oxygen–neon) are normally obscured by layers of helium and hydrogen. Consequently, the fraction of stellar mergers on the Q-branch and the details of their past evolution remain uncertain.

The most direct observational evidence of a stellar merger history comes from the surface composition. Among ultra-massive Q-branch white dwarfs, some have apparently typical hydrogen atmospheres, but others exhibit unusually carbon-enriched atmospheres^3,7^. Among these are six white dwarfs that have mixed hydrogen–carbon (DAQ type) atmospheres with number abundance ratios of log(C/H) = −0.5 to 0.97 (refs. ^8–10^). The photospheric carbon in all six systems has been detected in optical spectra. These rare objects have effective temperatures ranging from 13,000 to 17,000 K and masses between 1.13 and 1.19 *M*_⊙_, placing them firmly on the Q-branch (Fig. 1). Their observed atmospheric compositions can only be accounted for by extremely thin hydrogen and helium layers, where underlying carbon is dredged up by a superficial convection zone^11,12^. The thin hydrogen and helium layers, coupled with the large space velocity dispersions, strongly suggest a merger origin^8,9^.

**Fig. 2:**
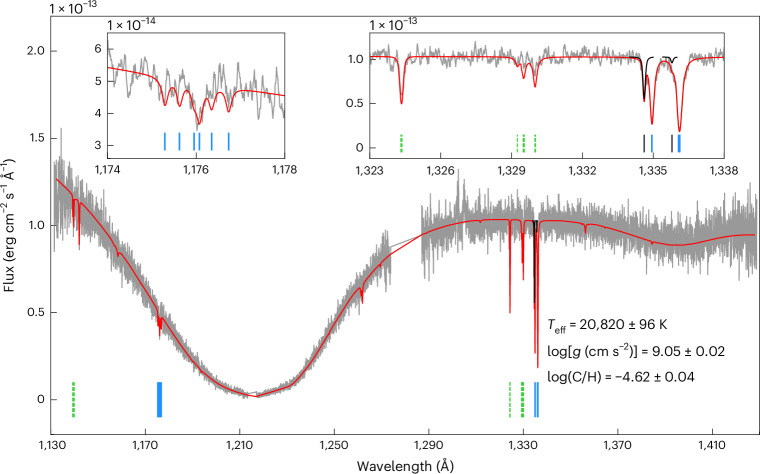
HST COS UV spectrum of WD 0525+526. The observed spectrum is shown in grey, with the best-fit model in red. The broad Lyman α absorption corroborates the hydrogen-rich atmosphere of WD 0525+526. Multiple narrow absorption lines of carbon are detected. The stronger transitions of carbon included in the fit are indicated by solid blue lines, and weaker ones or those with inaccurate atomic data that were excluded from the fit are shown by dashed green lines. The best-fit parameters obtained from the model fit are listed where *g* is the surface gravity of the white dwarf. The insets provide close-ups of the C iii (left) and C ii (right) lines that were used to determine the photospheric carbon abundance. The strongest C ii lines were used to determine the radial velocity of the white dwarf, which is +92.8 ± 0.6 km s^−1^. The black curves show the best-fit Gaussian profiles to the carbon absorption features originating from interstellar clouds (indicated by solid black lines) traversing at an average velocity of +18.7 ± 1.1 km s^−1^ along the line of sight, which is consistent with the predicted velocity of absorption (~19.1 km s^−1^) from the local ISM^69^.

Investigating the photospheric metal features present in the far-UV spectra of 311 hydrogen-atmosphere white dwarfs observed with the Cosmic Origin Spectrograph (COS) onboard the Hubble Space Telescope (HST)^13^, we detected photospheric carbon in WD 0525+526, a white dwarf located at the high-mass end of the Q-branch (Fig. 1). In addition to the broad Lyman α absorption in the spectrum confirming the atmosphere’s hydrogen-dominated nature^14^, WD 0525+526 exhibits strong absorption lines of atomic carbon (Fig. 2). The strongest carbon transitions are C ii at 1,334 and 1,335 Å, which are blended with absorption arising from the interstellar medium (ISM) along the line of sight. However, the presence of higher-order atomic transitions of carbon (C iii multiplet at 1,174–1,177 ) confirms that the red-shifted C ii lines at 92.8 km s^−1^ are of photospheric origin.

**Extended Data Fig. 1:**
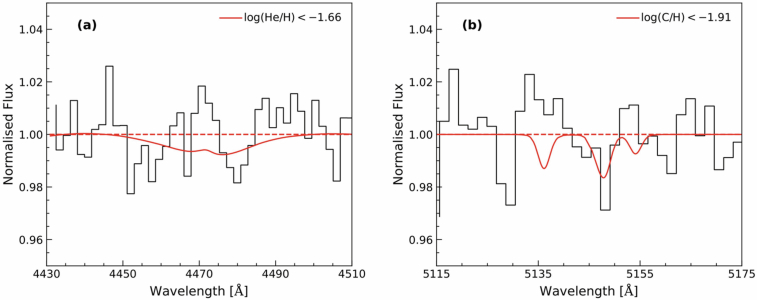
Optical upper limits on the helium and carbon abundances of WD 0525 + 526. The left (**a**) and right (**b**) panels illustrate the upper limit determination for helium and carbon, respectively, using the optical spectroscopic data^14^ shown as solid black lines. The model spectra corresponding to the 99th percentile upper limits are over-plotted as solid red lines. To calculate the upper limits, we created a grid of models with helium abundances log(He/H) varying from − 4.0 to − 0.5 and carbon abundances log(C/H) from − 8.0 to − 1.0 in steps of 0.05. For helium, we selected the spectral region 4430–4510 Å which contains a strong absorption feature from the He i line at 4472.75 Å. For carbon, we chose the spectral region spanning 5115–5175 Å with the strongest C ii lines from 5134.38 Å to 5152.52 Å. Fixing the *T*_eff_, $\log g$, and velocity to the best-fit values obtained from ultraviolet spectroscopy, the upper limits were determined following the same procedure as for a previously identified DAQ white dwarf^8^.

By fitting white dwarf atmospheric models^15^ to the flux-calibrated COS spectrum and the Gaia parallax^13^ and using a mass–radius relation^5^, we found an effective temperature of 20,820 ± 96 K and a mass of 1.20 ± 0.01 *M*_⊙_, confirming the white dwarf’s ultra-massive nature. Our parameters are consistent within ~3*σ* with the values reported from optical observations^14,16,17^. Keeping the white dwarf temperature and mass fixed, we modelled the carbon absorption lines with atmospheric models including the contribution of the interstellar lines and convolved with the COS line-spread function to derive a photospheric abundance of log(C/H) = –4.62 ± 0.04. The best-fitting model is shown in Fig. 2, and the white dwarf parameters are provided in Table 1. We retrieved the optical spectrum of WD 0525+526 (ref. ^14^) from the Montreal White Dwarf Database^18^; neither helium nor carbon lines were detected, yielding upper limits of log(He/H) < –1.66 and log(C/H) < –1.91 (both 99% confidence; Extended Data Fig. 1).

**Table 1:** Astrometric and spectroscopic parameters of WD 0525+526

Parameter	Value
Astrometric	
Right ascension (RA) (J2016) (deg)	82.46206
Declination (Dec) (J2016) (deg)	52.66239
Gaia Source ID	263082591016645504
Parallax (mas)	25.56 ± 0.05
Distance (pc)	39.12 ± 0.08
* μ*_*α*_ (mas yr^−1^)	+364.193 ± 0.046
* μ*_*δ*_ (mas yr^−1^)	−548.306 ± 0.034
Spectroscopic	
* T*_eff_ (K)	20,820 ± 96
log*g* (cm s^−2^)	9.05 ± 0.02
Radius (×10^−3^ *R*_⊙_)	5.44 ± 0.11
Mass (*M*_⊙_)	1.20 ± 0.01
* v*_l_ (km s^−1^)	+92.8 ± 0.6
* v*_ISM_ (km s^−1^)	+18.7 ± 1.1
log(C/H)	−4.62 ± 0.04
log (He/H)	<−1.66

The astrometric data are from Gaia DR3 at epoch J2016^19^. The COS spectroscopic parameters are based on a mass–radius relation considering a carbon–oxygen core and thin helium and hydrogen layers^5^. The errors represent 1*σ* statistical uncertainties in the measurements. Here, *μ*_*α*_ is the proper motion in RA, *μ*_*δ*_ is the proper motion in Dec, *R*_⊙_ is the radius of the Sun, *v*_l_ is the line-of-sight photospheric velocity not corrected for gravitational redshift, and *v*_ISM_ is the line-of-sight velocity of the interstellar cloud. The upper limit on the helium abundance was obtained from the optical spectrum^14^.

We conclude that WD 0525+526 is part of the small class of DAQ white dwarfs^9^, among which it is the hottest and nearest (distance = 39.1 ± 0.08 pc; ref. ^19^) member. It also has the lowest carbon abundance, ≃4–5 dex lower than that of the six cooler DAQ stars (Fig. 1). The detection of carbon in WD 0525+526 was only possible because of the COS spectroscopy available for this star. Whereas metal transitions in the optical become weaker with increasing temperature, the far-UV is rich in strong metal lines^20^, allowing the detection of small amounts of photospheric metals. The identification of WD 0525+526 as a DAQ among ≃20 white dwarfs with masses >1.1 *M*_⊙_ in the ≃1,000-strong spectroscopically complete 40-pc sample^21^ suggests that atmospheric carbon pollution of hot ultra-massive hydrogen-rich white dwarfs may be relatively common.

The previously published DAQ white dwarfs are thought to have a convective envelope in which the elements are homogeneously mixed, indicating that the detected carbon has been dredged up from the interior^8,9^. In contrast, the hot hydrogen-dominated atmosphere of WD 0525+526 is expected to be stable to convection^22^. This suggests a stratified structure in which an extremely thin hydrogen-rich layer floats atop a carbon-rich envelope, the latter providing the detected atmospheric carbon through upward chemical diffusion. However, this interpretation seems to lead to a contradiction: the carbon-rich envelope should be convective^23^ and the overshooting flows should efficiently mix carbon up to the surface^24^, thereby precluding the existence of a hydrogen-rich atmosphere.

To gain further insight into this conundrum, we modelled the distribution of the chemical elements as a function of depth in the envelope of WD 0525+526 using the STELUM code^25^. In what follows, the radial chemical profile is expressed as the elemental abundances as a function of logarithmic mass depth, log*q* ≡ log(1 − *m*_*r*_/*M*_WD_) (where *m*_*r*_ denotes the mass within radius *r* and *M*_WD_ the total mass of the white dwarf). We computed a static envelope model with a self-consistent chemical profile obtained by considering atomic diffusion and convective mixing in equilibrium. We assumed the atmospheric parameters inferred above, including surface abundances log(He/H) = −1.66 and log(C/H) = −4.62. We computed the chemical profile from the atmosphere inwards by either integrating the diffusive equilibrium equations in convectively stable regions or imposing complete mixing in convectively unstable regions^12^, initially ignoring convective overshoot. Under these assumptions, the observed surface composition uniquely determines the entire envelope composition (Methods).

**Fig. 3:**
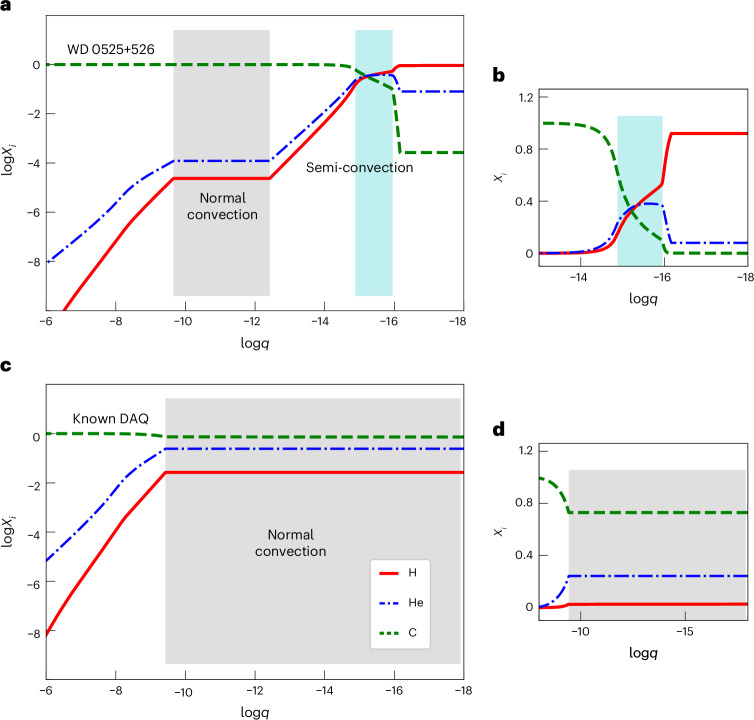
Envelope chemical structure of WD 0525+526 and a known DAQ white dwarf. The radial chemical profile of the model envelopes is expressed as the elemental mass fractions (*X*_*i*_, for element *i*) as a function of log*q* ≡ log(1 − *m*_*r*_/*M*_WD_). **a**, Chemical profile of the hot DAQ WD 0525+526 assuming the atmospheric parameters determined from UV spectroscopy (Fig. 2). **b**, The same as **a** but with emphasis on the outermost layers. **c**, Chemical profile of the cooler DAQ WD J2340−1819 assuming the atmospheric parameters^9^ *T*_eff_ = 15,836 K, log*g* = 8.95 and log(C/H) = +0.36, which are representative of the six known DAQ white dwarfs. **d**, The same as **c** but with emphasis on the outermost layers. In all panels, normal convection zones are shaded in grey, while semi-convection zones are shaded in blue. In the case of WD 0525+526, the normal convection zone with a uniform composition is much smaller, and there is a semi-convection zone showing a composition gradient near the surface. We used photospheric helium abundances corresponding to the upper limits determined from optical spectroscopy The legend in **c** applies to all panels.

**Extended Data Fig. 2:**
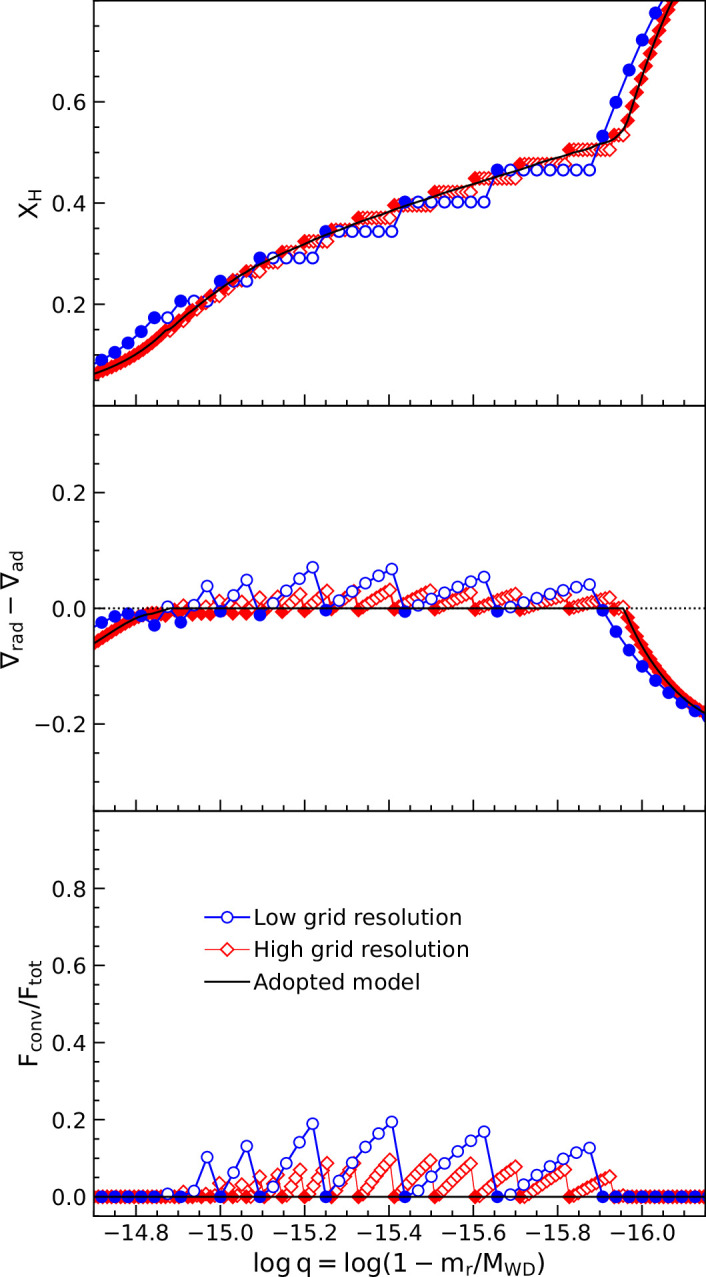
Properties of the semi-convective region in envelope models with different grid resolutions. The top, middle, and bottom panels show the hydrogen mass fraction, the difference between the radiative and adiabatic temperature gradients, and the ratio of convective to total energy flux, respectively, as a function of logarithmic mass depth. Illustrative models with low and high grid resolutions (≃ 35 and 100 grid points in the region of interest) are displayed as blue circles and red diamonds, respectively. Each grid point is depicted as an individual symbol, with empty and filled symbols denoting convective and non-convective grid points, respectively. The solid black lines show our adopted model, which was obtained by adjusting the chemical profile of the high-resolution model to enforce ∇_rad_ = ∇_ad_ in the semi-convective region. In the middle panel, the dotted black line indicates ∇_rad_ = ∇_ad_.

Our model (Fig. 3) confirms that the atmosphere of WD 0525+526 is not convective, allowing a thin hydrogen–helium shell to float on the surface and thus accounting for the low photospheric carbon abundance compared with the cooler DAQ stars. As anticipated, some convective mixing does occur just below the atmosphere (log*q* between about −15 and −16, shaded in blue in Fig. 3), but we found that this is inefficient mixing owing to semi-convection, rather than the usual efficient mixing associated with regular convection. Semi-convection occurs when a convective instability is strongly inhibited by a composition gradient (here, the hydrogen abundance gradient), leading to partial (rather than complete) mixing^26^. Although we did not include semi-convection explicitly in our modelling, it arises naturally in the form of alternating convective and non-convective layers in this region. The net result is that the slope of the abundance profiles falls between the limiting cases of diffusive equilibrium (steeper profiles) and uniform mixing (flat profiles), which is the signature of semi-convection^26^ (Methods and Extended Data Fig. 2). The occurrence of this process in white dwarfs with extremely thin hydrogen shells was suggested in 2007^27,28^, but here it is formally predicted in a detailed envelope model.

**Extended Data Fig. 3:**
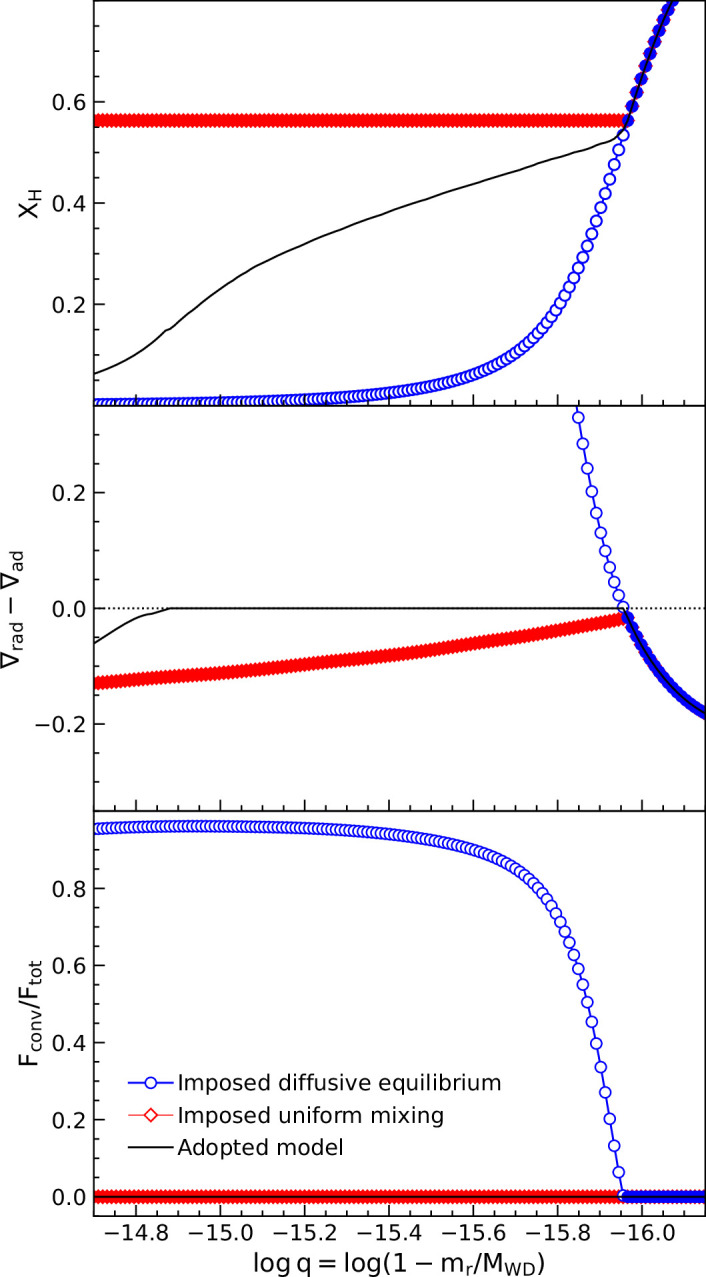
Properties of the semi-convective region in envelope models with imposed chemical profiles. The top, middle, and bottom panels show the hydrogen mass fraction, the difference between the radiative and adiabatic temperature gradients, and the ratio of convective to total energy flux, respectively, as a function of logarithmic mass depth. Illustrative high-resolution models with imposed diffusive equilibrium and imposed uniform mixing in the semi-convective region are displayed as blue circles and red diamonds, respectively. Each grid point is depicted as an individual symbol, with empty and filled symbols denoting convective and non-convective grid points, respectively. The model assuming diffusive equilibrium is convectively unstable, while the model assuming uniform mixing is convectively stable, therefore both models are inconsistent. The solid black lines show our adopted model, which is the only self-consistent solution.

**Extended Data Fig. 4:**
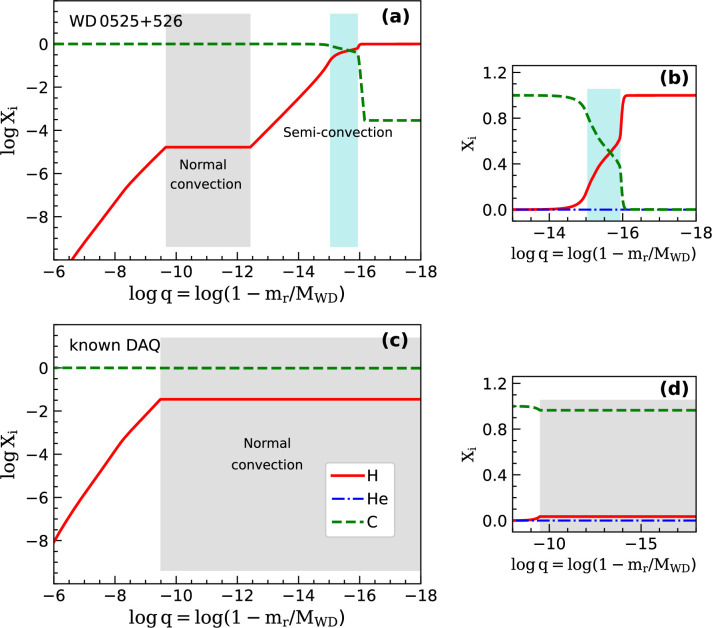
Envelope chemical structure of DAQ white dwarfs without any helium. The radial chemical profile of the model envelopes is expressed as the run of the elemental mass fractions with logarithmic mass depth, $\log q\equiv \log (1-{m}_{r}/{M}_{{\rm{WD}}})$. The top panels (**a** and **b**) show the chemical profile of the hot DAQ WD 0525 + 526 assuming the atmospheric parameters determined from ultraviolet spectroscopy (see Fig. 2). The bottom panels (**c** and **d**) show the chemical profile of the cooler DAQ WD J2340-1819 assuming the atmospheric parameters^9^*T*_eff_ = 15 836 K, $\log g=8.95$, and log(C/H) = + 0.36, which are representative of the six known DAQ white dwarfs. Normal convection zones are shaded in grey, while semi-convection zones are shaded in blue. These models do not include any photospheric helium.

We note that a standard carbon-driven convection zone^23^ generates additional mixing in a deeper region of the envelope (log*q* between about −10 and − 12, shaded in grey in Fig. 3). As a final step, we refined our model by including convective overshoot over one pressure scale height above and below this convection zone^24^, thereby slightly extending the mixed region (this is the final model shown in Fig. 3). We did not include overshoot beyond the semi-convection zone, as appropriate for such weakened convective flows. We performed a test calculation where we imposed a uniform composition in the semi-convective region, as would be expected for efficient mixing from regular convection and overshoot, and found that the resulting model is physically inconsistent (Methods and Extended Data Fig. 3). We interpret the outcome of this test as strong evidence for inefficient mixing owing to semi-convection in WD 0525+526, which effectively solves the apparent contradiction described earlier. Given that we could determine only an upper limit on the photospheric helium abundance, we also computed an envelope model without any helium, which yielded a similar chemical profile (Extended Data Fig. 4).

The total mass of hydrogen contained in our model of WD 0525+526 is 10^−13.8^ *M*_WD_. Similarly, the upper limit on the total mass of helium (that is, assuming the maximum surface abundance allowed by the observations) is 10^−12.6^ *M*_WD_. Both values are many orders of magnitude lower than expected from single-star evolution^29^ and thus indicative of a merger history, where thermonuclear burning during and after the merger event has depleted the hydrogen and helium contents^6,30^. For comparison, we also computed an envelope structure representative of the cooler and less massive DAQ stars (using the atmospheric parameters of WD J2340−1819 (ref. ^9^) as a typical example; Fig. 3), which yielded a homogeneous outer chemical profile^8^ (log*q* ≲ −10), as expected. The total hydrogen and helium masses of this model are 10^−10.6^ *M*_WD_ and 10^−9.3^ *M*_WD_, respectively. Therefore, WD 0525+526 is not only hotter and more massive than other DAQ stars, but also seems to have much lower residual hydrogen and helium contents. This indicates that higher-mass mergers may lead to more severe hydrogen and helium deficiencies.

We reviewed alternative scenarios that could account for the peculiar atmospheric composition of WD 0525+526 and found that none of them is compelling (Methods), leaving a merger as the most plausible explanation. Furthermore, the merger interpretation is independently supported by the location of WD 0525+526 and other DAQ stars on the Gaia Q-branch. Previous studies have shown that the delayed ultra-massive white dwarfs forming the Q-branch must have carbon–oxygen cores^5,31^. As massive single-star evolution produces white dwarfs with oxygen–neon cores^32^ (albeit with possible exceptions^30^), these objects are probably merger products^6,33^.

To better constrain the merger scenario, we investigated the three-dimensional space motions of WD 0525+526. The line-of-sight velocity determined from carbon lines in the UV spectrum is +92.8 ± 0.6 km s^−1^. Correcting for the gravitational redshift of 141.4 km s^−1^, based on the measured stellar mass and radius (Table 1), results in a radial velocity of −48.6 ± 0.6 km s^−1^. Combined with the Gaia proper motions, the Galactic space velocities are (*U*,*V*,*W*) = (− 8.1,−117.9,1.2) km s^−1^, corrected to the local standard of rest^34^. While kinematical ages are only constrained via velocity dispersions of statistical samples, the total space velocity of WD 0525+526 is unusually high for stars in the local volume^35^, including white dwarfs^36^, and notably lags behind Galactic rotation. Given that single stars cannot be assigned to any population with absolute confidence, the relevant probabilities (that is, whether a given star belongs to a thin disk, thick disk or stellar halo) suggest that WD 0525+526 is a thick disk star; it is 280 times more likely to belong to the thick disk population than the halo population^37^. This is nevertheless consistent with a star that can be as old as the disk itself, and also compatible with the other members of the DAQ spectral class^9^. Because the estimated total age under the assumption of single-star evolution is well under 1 Gyr (ref. ^38^), the space velocity supports a merger origin.

In its past evolution, WD 0525+526 probably exhibited an extremely hot, carbon-dominated atmosphere similar to that of the well-studied massive pre-white dwarf H1504+65 (ref. ^39^). The star then cooled down and atomic diffusion began to operate, causing residual hydrogen to float towards the surface^40,41^ and form the thin hydrogen-dominated, carbon-polluted layer that we observe today. As WD 0525+526 cools further, it will develop efficient convection in its outer envelope, thoroughly diluting the thin hydrogen layer and dredging considerable carbon back to the surface, and will therefore turn into a DQ-type white dwarf^9,12^. However, this transformation may not take place for several billion years, as the current cooling of WD 0525+526 is probably slowed down by the energy release of the ^22^Ne distillation process^5^. Our study highlights that only UV spectroscopy may be capable of identifying hotter DAQ merger remnants, thereby improving our understanding of this important channel of binary evolution.

## Methods

### Optical spectroscopy

In an attempt to better constrain the upper limit on the photospheric helium abundance, we acquired optical spectroscopy with the Binospec spectrograph^42^ at the MMT Observatory as part of our programme of spectroscopic follow-up of white dwarfs in the solar neighbourhood (PI: T.C.). The MMT/Binospec observations were carried out in September 2024, making use of the long slit with a width of 1 arcsec. We adopted the LP3500 filter, a grating with 1,000 lines per millimetre and a central wavelength of 4,500 Å. This set-up offered sensitivity at wavelengths ranging from 3,800–5,200 Å with a spectral resolution of *R* ≈ 3,900. Our observations consist of three consecutive exposures with individual exposure times of 1,200 s, yielding a total exposure time of 60 min. The Binospec data were reduced using the official IDL pipeline^43^. We determined the helium and carbon upper limits following the same procedure explained in Extended Data Fig. 1. The upper limits (log(He/H) < −1.5 and log(C/H) < −2.3 at 99% confidence) were found to be consistent with those based on the optical spectrum^14^ available from the Montreal White Dwarf Database.

### Envelope modelling

We used the STELUM code^25^ to compute static envelope models for WD 0525+526. We developed and exploited a new capability to produce equilibrium models in which the thermodynamic structure and chemical profile account for diffusion and convection in a self-consistent way. Such static equilibrium models are appropriate for our purpose given that the diffusive and convective timescales are both much shorter than the evolutionary timescale in the outer layers of white dwarfs^44,45^. We first obtained an approximate initial model by solving the stellar structure equations for a uniform composition (that is, imposing the measured surface abundances everywhere in the envelope). Holding the thermodynamic structure fixed, we then computed a detailed chemical profile, starting from the base of the atmosphere (Rosseland optical depth of 100, corresponding to log*q* ≃ −16.2) and proceeding inwards grid point by grid point. For convectively stable grid points, we determined the elemental abundances by integrating the diffusive equilibrium equations^12^. For convectively unstable grid points, we assumed complete mixing and thus set the same composition as the grid point immediately above. We treated convection using the Schwarzschild criterion and the ML2 version of the mixing-length theory^46^. The new chemical profile was then used to update the thermodynamic structure (and hence convective region), which in turn was used to recompute the chemical profile, with further iterations until convergence on a fully consistent model. The envelope integrations were performed down to log*q* = −3.0, which is located well inside the pure-carbon region.

We computed models for two possible atmospheric compositions, one with helium at the estimated upper limit and one without any helium. These two assumptions led to qualitatively similar chemical profiles (shown in Fig. 3 and Extended Data Fig. 4, respectively), and thus we focus hereafter on the model with helium. A convection zone is present at −12 ≲ log*q* ≲ −10 owing to the partial K-shell ionization of carbon^23^, producing flat abundance profiles. Convection also arises closer to the atmosphere at −16 ≲ log*q* ≲ −15 owing to the partial L-shell ionization of helium and carbon^23,47^, but in this case the convective grid points are interspersed with non-convective grid points, resulting in non-zero abundance gradients. We interpret this behaviour as the manifestation of semi-convection^26^ in our simple equilibrium model.

To support our interpretation, Extended Data Fig. 2 displays the radial profiles of key physical quantities in the region of interest; the panels show, from top to bottom, the hydrogen mass fraction *X*_H_, the difference between the radiative and adiabatic temperature gradients, ∇_rad_ − ∇_ad_, and the ratio of convective to total energy flux, *F*_conv_/*F*_tot_. For purely illustrative purposes, we first discuss a low-resolution model with only ≃35 grid points in the range −16 ≲ log*q* ≲ −15, shown as blue circles. Each grid point is depicted either as a filled symbol if it is convectively stable (∇_rad_ − ∇_ad_ < 0, *F*_conv_/*F*_tot_ = 0) or as an empty symbol if it is convectively unstable (∇_rad_ − ∇_ad_ > 0, *F*_conv_/*F*_tot_ > 0). At log*q* ≲ −15.9, the envelope is stable, so the hydrogen abundance decreases inwards as a result of diffusive equilibrium. The gradual enrichment in partially ionized helium and carbon (at the expense of fully ionized hydrogen) causes an increase in radiative opacity and thus in ∇_rad_. At some point, the latter becomes larger than ∇_ad_, implying that the plasma at this depth is convective and therefore that the composition remains constant on the next underlying grid points, given our assumption that convection generates uniform mixing. Proceeding farther inwards, the increase in temperature at constant composition translates into a gradual decrease in opacity and ∇_rad_ (a well-known feature of stellar envelopes). The latter eventually drops back below ∇_ad_, so the corresponding grid point is convectively stable and the composition is once again allowed to vary following diffusive equilibrium. However, the decrease in hydrogen abundance over the next grid point immediately restores the condition of convective instability and thus chemical mixing. This pattern of alternating convective and non-convective layers repeats itself until log*q* ≃ −14.9, where the hydrogen content becomes too low to notably affect the opacity. From this point inwards, ∇_rad_ monotonically decreases and thus the plasma remains stable.

The net result of these oscillations in ∇_rad_ − ∇_ad_ around 0 is a staircase-like chemical profile, with small discontinuities at non-convective grid points. These discontinuities are probably not physical: they are due to the finite number of grid points in our model as well as our assumption of complete mixing in convective regions. The latter hypothesis is probably inappropriate here given that the plasma is just on the edge between stability and instability. The same can be said of the blind use of the mixing-length formalism to calculate the convective flux and velocity, which abruptly vanish at non-convective grid points given the strictly local nature of the theory. Still, the emergence of a staircase-like chemical profile is informative as it constitutes a well-known signature of semi-convection in one-dimensional stellar models^48,49^ and even in three-dimensional hydrodynamical simulations^50^. To better capture the true chemical profile, we computed models with higher grid resolutions and found that the discontinuities became smaller as the number of grid points was increased. A model with ≃100 grid points in the region of interest, a threefold increase in resolution compared with the illustrative model discussed above, is displayed as red diamonds in Extended Data Fig. 2. To remove the remaining unphysical discontinuities, the abundance profiles of this model were smoothed by imposing strict convective neutrality, ∇_rad_ = ∇_ad_, resulting in the black lines in Extended Data Fig. 2. This corresponds to our adopted chemical profile, shown in Fig. 3 (and similarly in Extended Data Fig. 4 for the helium-free case). The outcome is a mild, continuous composition gradient in this region, intermediate between the much steeper gradient expected for pure diffusive equilibrium and the perfectly flat gradient expected for efficient convective mixing.

To further demonstrate that this is the sole physically viable solution, we performed numerical experiments where we forced the chemical profile in the region of interest to follow either one of the two limiting cases. These artificial models are shown in Extended Data Fig. 3, which is identical in format to Extended Data Fig. 2; the blue circles and red diamonds denote the cases of imposed diffusive equilibrium and uniform mixing, respectively. In the model assuming diffusive equilibrium, the hydrogen abundance sharply decreases inwards, such that ∇_rad_ becomes much larger than ∇_ad_, accordingly generating a convection zone that transports over 90% of the total energy flux. Such a vigorous convection zone should completely mix the various elements, and hence this model is clearly inconsistent. In the model assuming a uniform composition, the large hydrogen abundance causes ∇_rad_ to remain smaller than ∇_ad_ and thus entirely suppresses the convective instability, again leading to a contradiction. Therefore, the only self-consistent solution is an intermediate composition gradient such that the equality ∇_rad_ = ∇_ad_ is strictly satisfied, as in our adopted model. The physical interpretation is that this region experiences semi-convection: weak convective flows inducing just enough mixing to maintain convective neutrality^26^. This is a self-regulating process, as any deviation towards stronger or weaker mixing would drive the system back to equilibrium, thus leading to a unique chemical profile.

Finally, we note that our test where uniform mixing was imposed effectively rules out the possibility of mixing due to overshoot beyond the semi-convective zone. Indeed, adding overshoot in our model would shift the boundary of the mixed region closer to the surface and thus increase the hydrogen abundance in that region. This would in turn push the model farther from the condition of convective instability, worsening the contradiction described above. Therefore, we conclude that overshoot is negligible in this case, in line with the idea that fluid motions arising from semi-convection are considerably weaker than those characterizing normal convection. This justifies our choice to include overshoot solely beyond the standard convection zone at −12 ≲ log*q* ≲ −10.

In our envelope calculations, we placed the outer boundary at Rosseland optical depth of 100 corresponding to log*q* ≃ −16.2, implying that we imposed a uniform composition above that point, as can be seen in Fig. 3. We made this choice for consistency with the atmosphere models used in the spectroscopic analysis, which assume a homogeneous chemical mixture^15^. In reality, since the atmosphere is radiative, diffusion is expected to operate all the way out to the surface. To verify that the presence of the semi-convection zone is robust to this assumption, we also computed envelope models with outer boundaries at lower optical depths. We found that such models still contained a semi-convective region of similar extent, the only difference being that this region was slightly shifted upwards (approximately following Δlog*τ* ≈ Δlog*q*, where *τ* denotes the optical depth). Therefore, we conclude that this numerical parameter does not affect our conclusion regarding the occurrence of semi-convection in WD 0525+526. An improved analysis would require atmosphere models that similarly take diffusion into account and thus allow a stratified chemical profile^51,52^, but such models currently do not exist for hydrogen–carbon compositions and their development is beyond the scope of this Article.

### Alternative scenarios for the atmospheric composition

We interpreted the hydrogen-dominated, carbon-polluted atmosphere of WD 0525+526 as the result of severe hydrogen and helium deficiencies arising from a past stellar merger. In addition to this interpretation, we explored other scenarios that could potentially account for the photospheric carbon abundance observed in WD 0525+526.

The process of radiative levitation can support heavy elements (including carbon) at the surface of hot white dwarfs with *T*_eff_ ≳ 20,000 K (ref. ^53^). However, this mechanism is predicted to be ineffective at the relatively low temperature and very high surface gravity of WD 0525+526 (refs. ^54,55^).

Another possible source of photospheric carbon is accretion of material from the ISM^56^, a disrupted planetary system^54^ or a close (sub)stellar companion^57^. However, in all cases, other heavy elements would be detected alongside carbon in the UV spectrum of the white dwarf. In the case of planetary debris with a standard rocky composition (such as bulk Earth), silicon would be the most noticeable element. In the case of a volatile-rich object (such as a comet), other elements such as nitrogen, phosphorus and sulfur would be observed. For accretion from the ISM or a companion, all these species may be present. We did not detect photospheric silicon or any heavy element other than carbon in the COS spectrum of WD 0525+526. From the absence of the Si ii doublet near 1,265 Å, we measured an upper limit of log(Si/H) < −8.5, implying a C/Si ratio ≳3 orders of magnitude higher than that typically produced by accretion^54^. Furthermore, we searched for infrared excess and photometric variability that would indicate the presence of a debris disk or a close companion. We built the spectral energy distribution of WD 0525+526 using available optical and infrared photometry, including Gaia *G* − *G*_BP_ − *G*_RP_^19^, Panoramic Survey Telescope and Rapid Response System (Pan-STARRS) grizy^58^, and Wide-field Infrared Survey Explorer (WISE) W1^59^. We did not detect any anomalous excess in the WISE W1 band. We looked for photometric variability using Zwicky Transient Facility^60^ and Transiting Exoplanet Survey Satellite^61^ observations. We found that WD 0525+526 is not variable on timescales of a few minutes to several hours, with variability limits of at least 0.5% in Zwicky Transient Facility DR20 and 0.4% from about three months (Sectors 19, 59, and 73) of 2-min-cadence data from the Transiting Exoplanet Survey Satellite. Therefore, all variants of the accretion scenario seem highly unlikely.

Many cool white dwarfs with *T*_eff_ ≲ 10,000 K exhibit a helium-dominated, carbon-polluted atmosphere^7,62^ ascribed to dredge-up of internal carbon by a helium-driven convection zone^63,64^. These objects are believed to originate from single-star evolution involving a so-called late helium-shell flash, a phenomenon that drastically reduces the hydrogen content while leaving the helium content largely intact^63,65^. The carbon dredge-up process requires a deep convection zone and thus usually occurs at effective temperatures lower than that of WD 0525+526. There are a few exceptions, notably a handful of helium-rich white dwarfs with temperatures (*T*_eff_ ≃ 22,000–26,000 K) and carbon abundances (log(C/H) ≃ −5.5) similar to those of WD 0525+526 (while being devoid of other heavy elements)^66,67^. The cause of the carbon pollution in these stars is still debated but has been tentatively attributed to convective dredge-up^67^. In any case, these objects are notably different from the DAQ white dwarfs, as they have typical masses (~0.6 *M*_⊙_ on average) and helium-dominated atmospheres, and therefore they probably have a distinct origin^68^.

## Supplementary information

Supplementary Data 1 MMT/Binospec spectrum of WD 0525+526. Columns are: air wavelength (Å), flux (erg cm^−2^ s^−1^ Å^−1^), and flux error (erg cm^−2^ s^−1^ Å^−1^).

Supplementary Data 2 Best-fitting model spectrum for WD 0525+526 (Fig. 2) without convolution to COS spectral resolution. Columns are: vacuum wavelength (Å) and model flux (erg cm^−^^2^ s^−^^1^ Å^−^^1^).

Supplementary Data 3 Model envelope chemical profile of WD 0525+526 with helium at the upper limit (Fig. 3). Columns are: logarithm of mass depth (log*q*), logarithm of hydrogen (logH), helium (logHe) and carbon (logC) mass fractions, and a number indicating the presence and type of convective mixing, where 0 denotes no mixing, 1 denotes normal convection, 2 denotes overshoot and 3 denotes semi-convection.

Supplementary Data 4 Model envelope chemical profile of WD 0525+526 without any helium (Extended Data Fig. 4).

Supplementary Data 5 Model envelope chemical profile of WD J2340−1819 with helium at the upper limit (Fig. 3).

Supplementary Data 6 Model envelope chemical profile of WD J2340−1819 without any helium (Extended Data Fig. 4).

## Data Availability

The COS spectra of WD 0525+526 are publicly available in the MAST HST archive (https://mast.stsci.edu/search/ui/#/hst) under programme ID 15073. The MMT/Binospec spectrum, best-fitting model spectrum and chemical profile are provided in Supplementary Data 1–6.

## Code availability

The Koester model atmosphere code^15^ and the STELUM evolution code^25^ are not available as they require substantial expert training and the authors do not have the resources to support general public use. However, their associated references can be consulted for further details.
